# Tibial condylar valgus osteotomy (TCVO): Surgical technique and clinical results for knee osteoarthritis with varus deformity

**DOI:** 10.1016/j.jcot.2021.101589

**Published:** 2021-09-07

**Authors:** Tsukasa Teramoto, Shota Harada, Nobuyuki Takenaka, Takashi Matsushita

**Affiliations:** Department of Traumatology, Fukushima Medical University, Trauma & Reconstruction Center, Southern TOHOKU General Hospital, Japan

**Keywords:** Knee, Tibia, Tibial condylar valgus osteotomy, Knee osteoarthritis, Joint stabilized surgery, Joint instability

## Abstract

Tibial condylar valgus osteotomy (TCVO) is an intra-articular proximal tibial osteotomy developed in 1989 and has since been used for the treatment of knee osteoarthritis (OA) associated with genu varum. This article describes the surgical technique and clinical results of TCVO. TCVO can be used for all grades of varus knee OA in patients of any age. he preoperative range of movement should be at least 90°. Preoperative screening showed varus-valgus instability due to an intra-articular deformity of the proximal tibia. Using intraoperative image intensification, a sagittally oriented “L”-shaped osteotomy is made from the medial to the tibial tuberosity to the center of the tibial plateau between the medial and lateral tibial spines. The separation of the osteotomy using the lamina spreader is gradually increased using an image intensifier guidance until the articular surface of the lateral tibial plateau comes in contact with the articular surface of the lateral femoral condyle. Adequate correction is indicated by parallelism of the lateral tibial plateau and a line tangential to the distal convexity of the lateral femoral condyle on an anteroposterior (AP) image and the elimination of the valgus instability with the knee in extended position. A “T”-plate (locking or non-locking plate or circular external fixator) is used to fix the osteotomy in the corrected position. Synthetic or autologous bone grafts can be used. We used the Japanese Orthopaedic Association score to evaluate the patient's function and also measured the %MAD, medial plateau opening angle, medial plateau angle, and lateral plateau opening angle on an AP view of the long length roentgenogram of the lower limb (standing position). The JOA score, radiologically measured values, and instability of the knee joint remarkably improved.

## 1 Introduction

Surgical strategies, including high tibial osteotomy (HTO), UKA, and TKA, have been described for the treatment of knee osteoarthritis (OA). TKA is an accepted, reliable, and effective surgical procedure for end-stage OA patients to relieve pain, restore function, and improve quality of life. At present, TKA and UKA are widely performed for moderate or severe OA of the knee; however, the survival of any artificial joint is limited. Polyethylene-bearing wear is historically one of the most common causes of UKA and TKA failure.^14^ UKA and TKA are therefore poorly suited to young patients or those who will place high demands on their implants through heavy manual work or return to sporting activity. HTO is an accepted procedure for treating varus alignment of the knee associated with medial mechanical axis deviation, leading to medial compartment overload and subsequent OA. HTO is contraindicated in patients with severe OA of the medial compartment.^1^, ^2^, ^3^, ^4^, ^10^

We developed the tibial condylar valgus osteotomy (TCVO) and have used this technique since 1989 for moderate to severe varus knee OA. Intra-articular proximal tibial osteotomy achieves the objective of maintaining ROM, reducing pain, and restoring function, including high-demand physical activity. TCVO is a surgical technique that realigns the knee joint without fibular osteotomy. This article describes the surgical technique and clinical results of TCVO for knee OA with varus deformity.

## 2 Patients and methods

The study cohort comprised 145 patients (34 men and 111 women) with 171 knees with medial OA who were treated with TCVO. The average age was 66.3 years. According to the Kellgren-Lawrence (K-L) classification for OA grade, 35 knees were classified as grade 2, 57 knees were classified as grade 3 and 79 knees as grade 4. TCVO was fixed using a conventional plate, locking plate and an Ilizarov external fixator. The average follow-up period was 5.4 years. We evaluated the clinical results using the Japanese Orthopaedic Association (JOA) scores. The validity and reliability of the JOA scores for osteoarthritic knees have already been reported.^9^

**Fig. 1:**
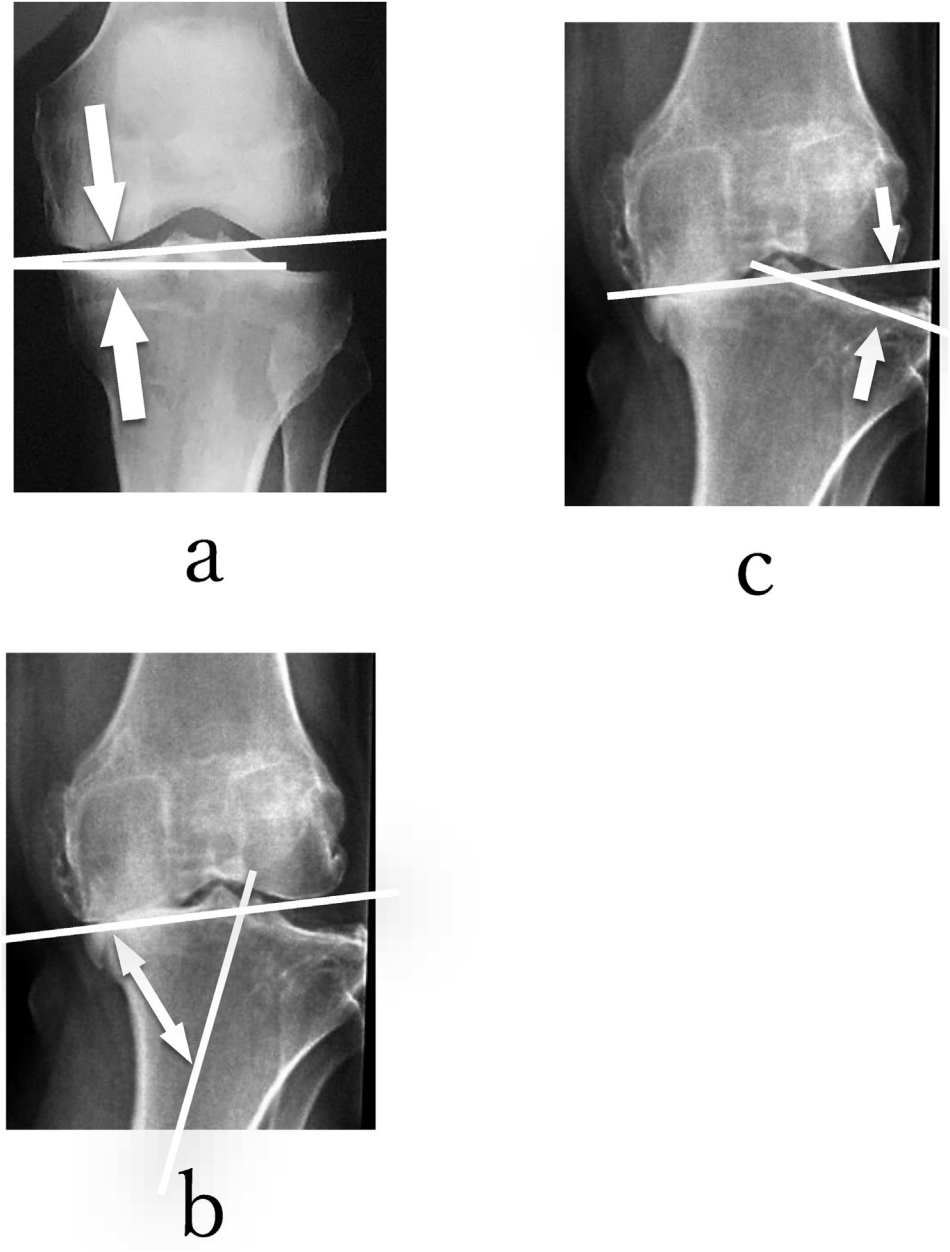
MPOA (a)was measured as the angle between the tangential line of both the medial femoral condyle and the lateral femoral condyle and the articular surface of the medial tibial plateau while excluding the osteophyte at the medial tibial plateau surface. MPA (b)was the medial angle between the articular surface of the medial plateau and the axis of the proximal part of the tibia. LPOA(c) was measured as the lateral opening angle between the tangential line of both the medial and lateral femoral condyles and the articular surface of the lateral tibial plateau. MPOA, MPA, and LPOA were measured while excluding the osteophyte at the tibial plateau surface. The medial opening angle of MPOA was positive, as well as the lateral opening angle of LPOA.

We used the JOA score for the patient's symptoms and function of the knee joint before and after the operation. On a 100-point system, points were allocated as follows: 30 points for pain and walking ability, 25 points for pain and stair walking ability, 35 points for flexion angle, and 10 points for swelling of the knee joint. An anteroposterior (AP) view of the long length roentgenogram of the lower limb (standing position) was performed for the radiological evaluation. The percentage of mechanical axis deviation (%MAD),^16^ medial plateau opening angle (MPOA), medial plateau angle (MPA), and lateral plateau opening angle (LPOA) were measured to evaluate the alignment and articular shape of the knee joint before and after TCVO. (Fig. 1) %MAD was defined as the ratio of the distance from the medial border of the proximal tibia to the mechanical axis of the lower limb to the width of the proximal tibia.^16^ The varus stress angle and valgus stress angle were measured as the angle between the tangential line of the medial femoral condyle and the lateral femoral condyle and the articular surface of the tibial plateau in varus and valgus stress radiographs under the image intensifier with the knee extended and flexed at 10° before and after TCVO. The total amplitude of the varus stress angle and valgus stress angle was identified as the knee joint instability angle (KJIA).

### 2.1 Clinical indications

Kellgren-Lawrence classified varus knee OA into four grades using weight-bearing radiographs. A simplification of the K-L classification^8^ describes grade 1 as doubtful, grade 2 as mild, grade 3 as moderate, and grade 4 as severe. HTO may be indicated for grade 2 or 3 OA; however, it is unsuitable for the treatment of grade 4 OA. HTO is also contraindicated in patients with tricompartmental OA or in large areas of exposed subchondral bone.^2^, ^4^, ^7^, ^11^ Kettlecamp reports that HTO is unsuitable for treating the “teeter” knee, where an intra-articular deformity exists with intercondylar angulation and varus-valgus instability. There are several other reports of poor results following HTO in patients with knee joint instability.^13^, ^14^, ^17^ In contrast, TCVO is indicated for all grades of varus knee OA in patients of any age with a knee ROM of at least 90°. It can be used to restore function and allow return to social, work, and sporting activities, independent of the patient's age or K-L grade. TCVO is contraindicated in patients with joint degeneration secondary to infection, rheumatoid arthritis, neuroarthropathy, or avascular necrosis.

### 2.2 Surgical technique

The procedure was performed under general anesthesia with the patient in the supine position. A pneumatic thigh tourniquet was used in this study. An examination under anesthesia was performed to assess knee joint stability to varus-valgus stress with the knee extended and flexed at 10°. A medial longitudinal parapatellar skin incision was marked just proximal to the level of the knee joint to approximately 5 cm distal to the tibial tuberosity. The length of the skin incision may vary according to the fixation method used.

The surgical approach is carried down to the subperiosteal plane, allowing the pes anserinus and medial collateral ligament to be elevated from the medial surface of the tibia. The elevation continues distally beyond the level of the planned exit point of the transverse portion of the osteotomy and posteriorly to the midsagittal point of the tibia.

The direction of the sagittal cut should be from the medial to the tibial tuberosity to the center of the knee joint between the medial and lateral tibial spines. The transverse part of the osteotomy connects the base of the sagittal osteotomy to the medial proximal tibia and creates an “L” shape. Separation of the osteotomy using the lamina spreader is gradually increased using an image intensifier guidance until the articular surface of the lateral tibial plateau comes in contact with the articular surface of the lateral femoral condyle. As a result of the elevation of the medial tibial plateau, the congruence of the knee joint is improved and the tibial spines are depressed relative to the intercondylar notch of the distal femur. This depression creates tension to the cruciate ligaments. If there is significant sinking of the lateral tibial plateau during this correction, a 1.8-mm subchondral K wire is introduced to control the elevation of medial tibial plateau above the level of lateral tibial plateau. The direction of the opening wedge is usually not exactly in the coronal plane and more often occurs in an oblique plane. When viewed from the end of the operating table, opening the osteotomy with the laminar spreader tends to produce an external rotation of the leg. This rotation should be created automatically through coaptation of the articular surfaces. This combination of oblique plane and rotational correction indicates that the TCVO corrects deformity in all three dimensions and is not simply a coronal plane correction. Osteophytes, meniscal pathology, or articular cartilage defects do not require treatment using this technique and would unnecessarily expose the knee joint.

Adequate correction is indicated by parallelism of the lateral tibial plateau and a line tangential to the most distal point of the convexity of the lateral femoral condyle on an AP view using the image intensifier. A critical factor when assessing adequacy of correction is the stability of the knee joint. The laminar spreader is used to gradually and repeatedly open the wedge until both tibial plateaus are in contact with the femoral condyles and the knee has become stable to varus-valgus stress. The mechanical axis of the lower limb and medial proximal tibial angle were not used to determine adequacy of correction.^12^, ^17^, ^19^

Stress imaging of the knee following correction confirmed that improvement in knee stability was achieved, which is the primary goal of the TCVO.

Persistent instability suggests inadequate correction, and the reason for this should be sought by further assessment. Inadequate medial soft tissue subperiosteal release, inadequate opening of the osteotomy, and fracture of the lateral intercondylar prominence at the time of osteotomy separation using the lamina spreader were the causes of failed correction. Fixation of the osteotomy can be achieved with a locking or non-locking “T”-plate or, alternatively, a circular external fixator (CEF). Synthetic or autologous bone grafts may be used to improve stability.

**Fig. 2:**
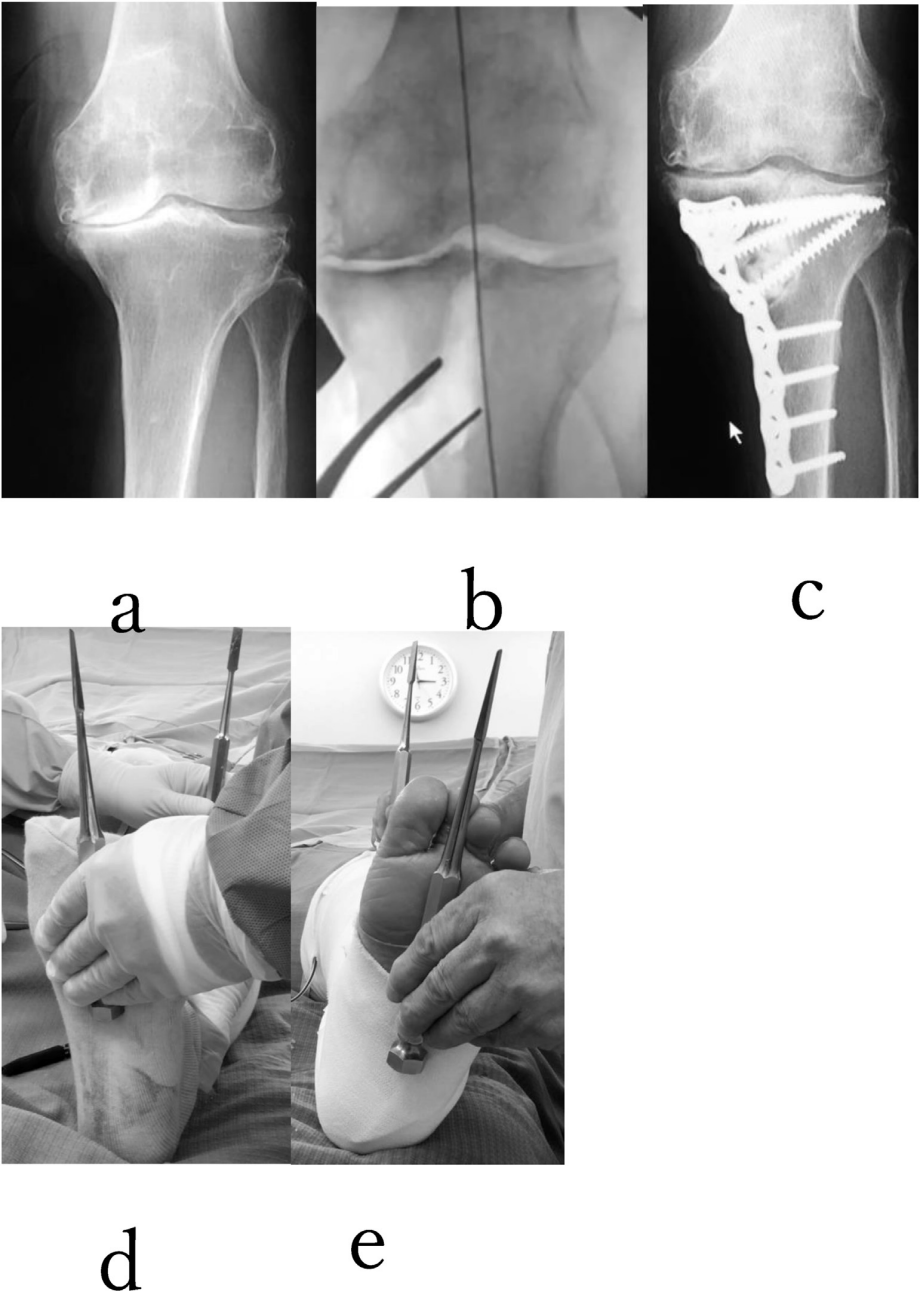
A 76-year-old woman with grade 4 (K–L) varus-type knee osteoarthritis in her left knee was treated with TCVO(a). After TCVO(b,c), opening the osteotomy with the laminar spreader produces an external rotation of the leg when viewed from the end of the operating table. (d, e) The JOA score was from 50 points to 85 points. The range of motion of his right knee was maintained postoperatively (0° extension, 95° flexion). She could walk and work as a farmer without pain. %MAD was from −8% to 50%(a, b, c). KJIA (knee flexed) was from 11.1° to 0° after TCVO.

**Fig. 3:**
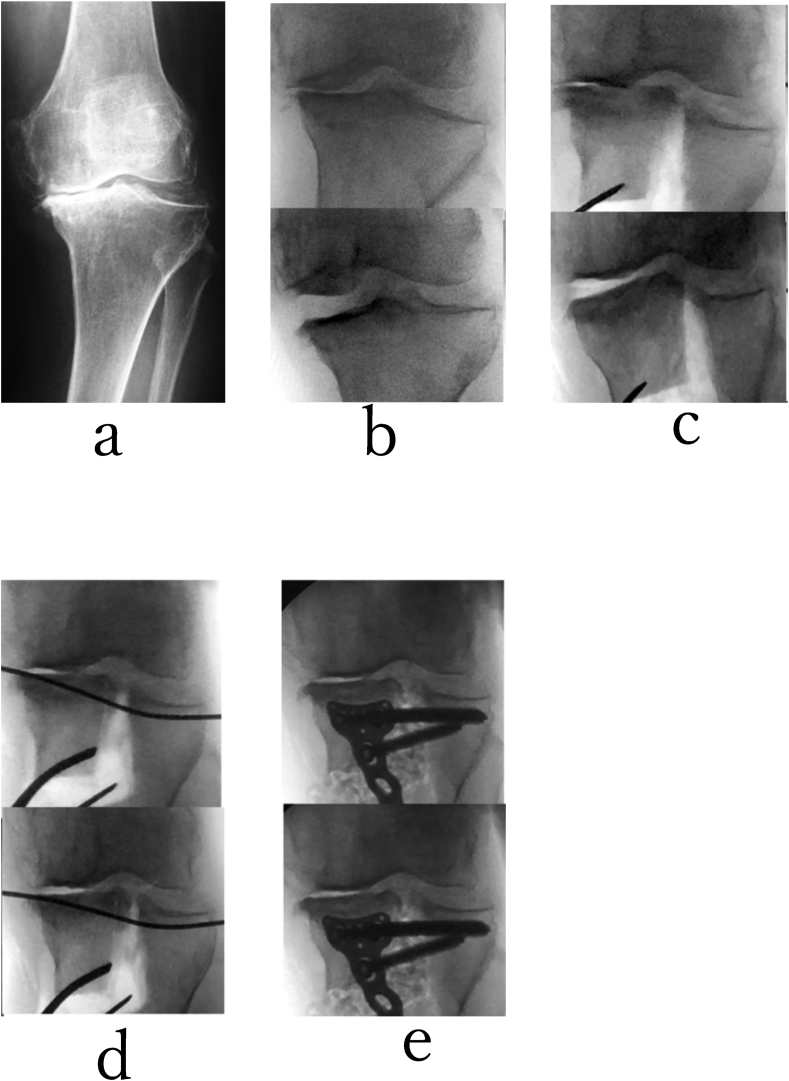
A 73-years-old woman with grade 4 (K–L) varus type knee osteoarthritis in her left knee were treated with TCVO(a). Varus stress (upper row) and valgus stress (lower row)with the knee extended were performed (b) under an image intensifier before TCVO, (c) after correction without a subchondral K wire, (d) after correction with a subchondral K wire, and (e) after TCVO. KJIA (knee extended position) was from 14.5°(b) to 8.5°after correction without a K wire(c), to 0°after correction with K wire (d), and to 0°after TCVO (e). A 1.8-mm subchondral K wire is introduced to control the elevation of medial tibial plateau above the level of lateral tibial plateau. Her left knee joint with the knee extended position was stabilized after correction with K wire and after TCVO(d,e).

The locking plate provides a fixed-angle construct and improves the stability of the fixation when compared with the non-locked plating; however, care must be taken during insertion of the proximal screws. The anteromedial application point of the plate can lead to the proximal locking screws being directed toward the popliteal fossa, and the surgeon should take care to avoid any penetration of the posterior cortex by either drill or screw. Non-locking plates allow greater directional control of the proximal screws at the expense of angular stability provided by a locking plate. Bone grafts are usually inserted using either an autologous iliac crest bone graft or a synthetic bone graft substitute such as β-tricalcium phosphate (β-TCP).

in cases where there is over 25 mm of separation between the bone fragments on the medial side of the tibia following correction, it may be preferable to use a CEF to reduce the risk of wound healing problems due to the added soft tissue tension with plating. Other indications for CEF include poor soft tissue quality, small osteotomy fragment due to significant varus intra-articular deformity, previous bone infection, and the need for a secondary extra-articular osteotomy (Fig. 2), (Fig. 3)

### 2.3 Postoperative rehabilitation

Active knee joint movement is encouraged, but passive knee ROM manipulation is not recommended until at least 8 weeks following surgery. WB on the operated limb is otherwise restricted until 3–6 weeks when partial weight bearing can begin depending on soft tissue status.^18^, ^19^ If a CEF is used, it is usually removed at around 3–4 months, depending on radiographic appearances.^20^ The patients returned to the usual heavy work and sport activity if they did not complain of knee joint pain. The average time required for the return to work in my patients is about 6 months. All patients have been done the thromboprophylaxis post operatively for 14 days along the guideline of Japan Orthopaedic association (JOA).

## 3 Results

The average JOA score was 45.3 ± 7.5 points to 81.1 ± 8.2 points after TCVO. The preoperative average of %MAD is from −6.8 ± 18.1% to 59.2 ± 10.3% after TCVO. The preoperative average of MPOA significantly decreased from 6.5° ± 2.4°–4.3° ± 3.0° after TCVO (p < 0.001). The preoperative average of MPA significantly decreased from 75.6° ± 3.4°–90.9° ± 4.3° after TCVO. The preoperative average of LPOA significantly decreased from 15.6° ± 3.4°–3.9° ± 2.1° after TCVO (p < 0.001). (Table 1) KJIA with the knee extended ranged from 2.7° ± 2.4°–0.44° ± 0.84°. (p < 0.001) KJIA with the knee flexed ranged from 6.1° ± 3.1°–1.4° ± 1.7°. (p < 0.001)- (Table 2) We have encountered minor complication of pin track infection when a circular frame was used. They responded to oral antibiotics and none of the patients needed pin removal or return to theatre for addition of another pin. These infections did not result in septic arthritis of the knee. There was one case of non-fatal pulmonary embolism. We haven't encountered non-union of the osteotomy.

**Table 1:** Changes of JOA score and the index of the long length roentgenogram of the lower limb before TCVO and at the time of follow up.

	Before DTOO	Follow up	P value^a^
JOA score	59 ± 9.4 points	83.2 ± 9.1 points	(p < 0.001)-
%MAD	10.4% ± 20.3% (−56.4% to 54.4%)	58.8% ± 11.3% (24.0%–88.80%)	(p < 0.001)--
LPOA	5.4° ± 7.0° (−15.0 °to 26.4°)	−7.1 ± 5.7° (−23.7°–12.3°)	(p < 0.001)--
MPA	81.5° ± 5.4° (63.8°–98.0°)	96.9° ± 5.1° (83.0°–114.0°)	(p < 0.001)--
MPOA	−1.9° ± 4.1° (−14.0°–12.3°)	−5.0° ± 5.6° (−24.1°–15.2°)	(p < 0.001)--

JOA score: Japanese Orthopaedic Association Score.

%MAD: Percentage of mechanical axis deviation.

LPOA:Lateral plateau opening angle.

MPA:Medial plateau angle.

MPOA:Medial plateau opening angle.

a Paired *t*-test.

**Table 2:** Changes of the knee joint instability angle before TCVO and just after TCVO.

	Before TCVO	Just after TCVO	P value^a^
KJIA (knee extended position)	2.7° ± 2.4°	0.44° ± 0.84°	(p < 0.001)-
KJIA (knee flexed position)	6.1° ± 3.1°	1.4° ± 1.7°	(p < 0.001)--

KJIA: Knee joint instability angle(The total amplitude of the varus stress angle and valgus stress angle was identified as the KJIA).

a Paired *t*-test.

### 3.1 Case presentation

**Fig. 4:**
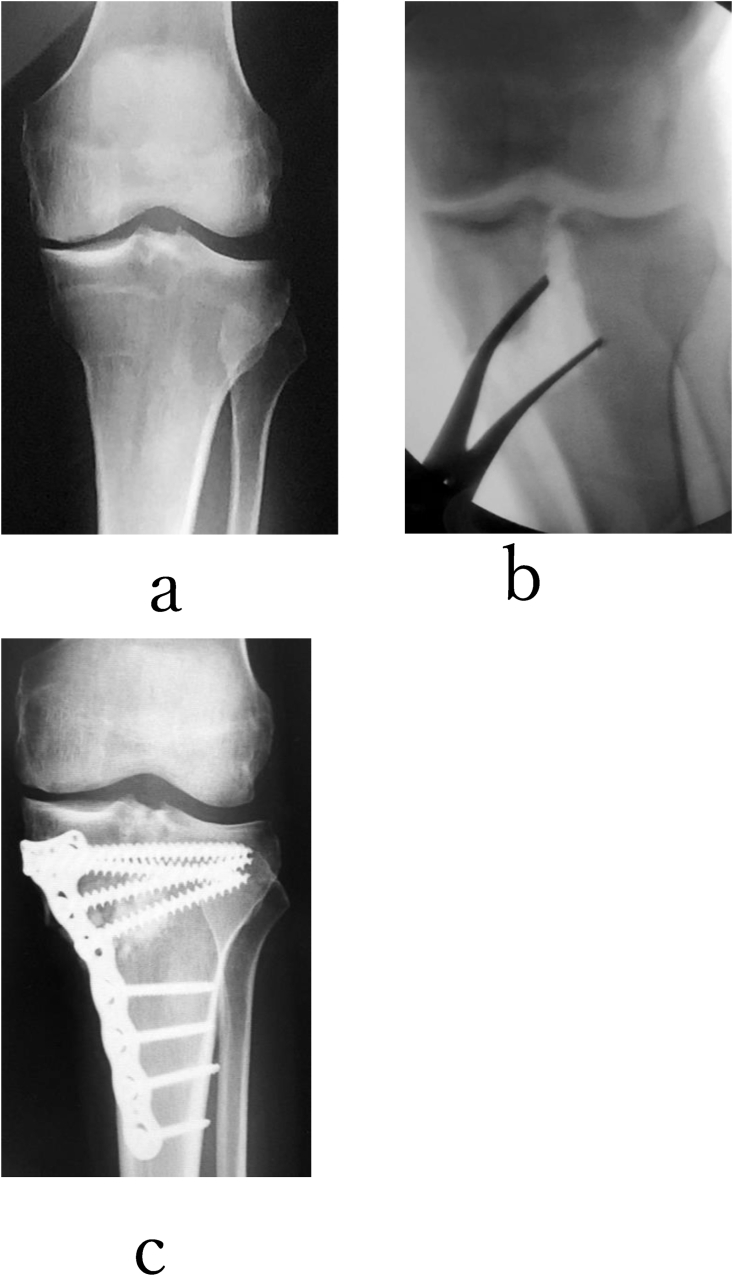
A 62-year-old man with grade 3 (K–L) varus-type knee osteoarthritis in his left knee was treated with TCV. Before TCVO, he complained the recurrent hydrosis of the left knee joint. He could not walk with two crutches. (a)TCVO was achieved using the non-locking T-plate(b,c,). Two years after TCVO, the recurrent hydrosis improved. He could return to work as a farmer. The JOA score was from 45 points to 85 points after TCVO. %MAD was from 45% to 75% after TCVO. KJIA (knee flexed) was from 8° to 0° after TCVO. His right knee joint was stabilized after TCVO.

**Fig. 5:**
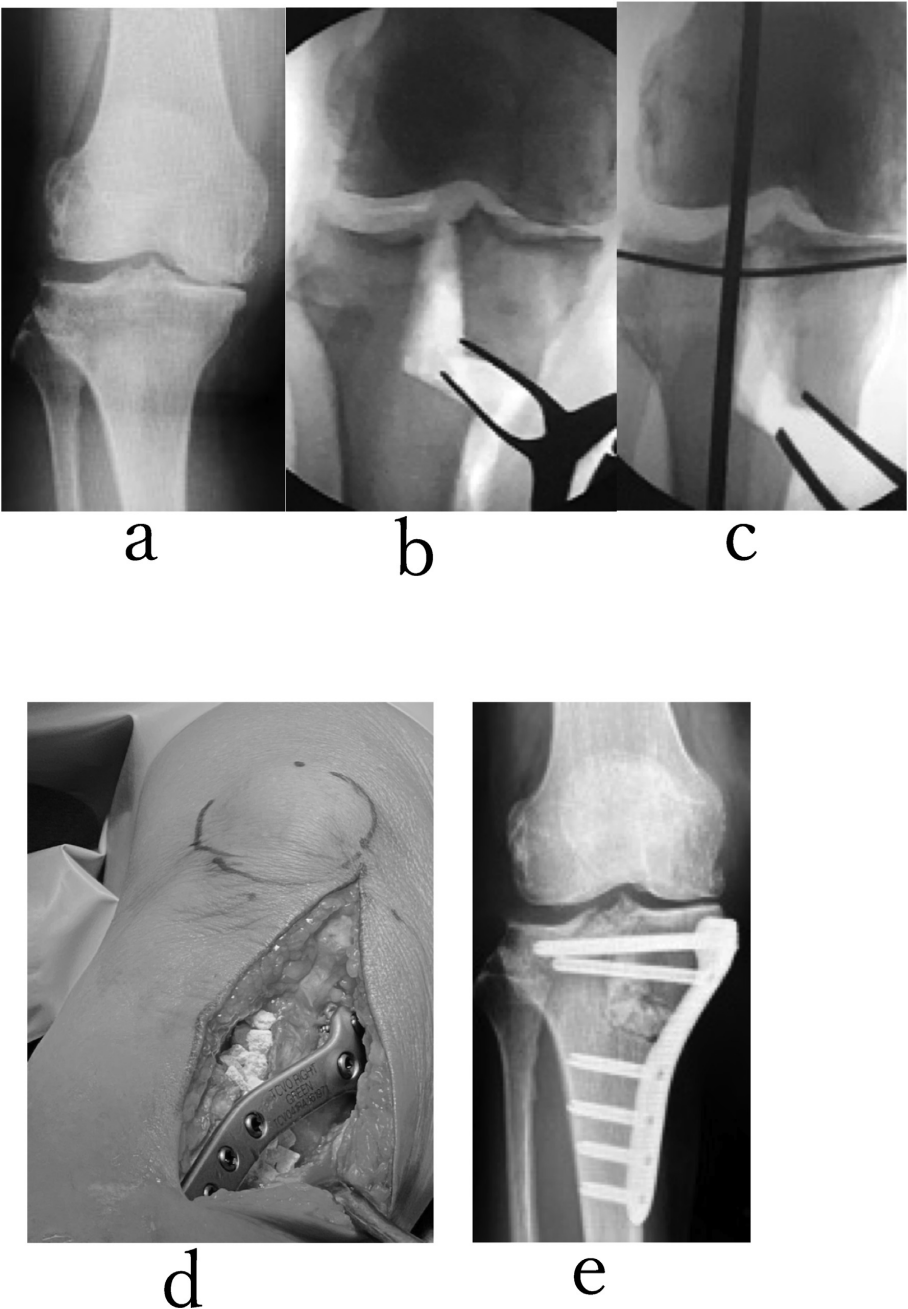
A 73-year-old man with grade 4 (K–L) varus-type knee osteoarthritis in his right knee was treated with TCVO. He could not walk without pain. (a) At first correction, there is sinking of the lateral tibial plateaus for the medial tibial plateau, and right knee joint instability was not improved after correction. (b) A 1.8-mm subchondral K wire is introduced to control the elevation of medial tibial plateau above the level of lateral tibial plateau. The sinking of the lateral tibial plateau and the instability of knee joint were decreased after correction. (c) Adequate correction was indicated by parallelism of the lateral tibial plateau and a line tangential to the distal most point of the convexity of the lateral femoral condyle on an AP view using an image intensifier. (d) Fixation of the osteotomy can be achieved with the locking plate and grafted β-TCP. (c, d) %MAD was from 10% to 55%, (a, c, e).

**Fig. 6:**
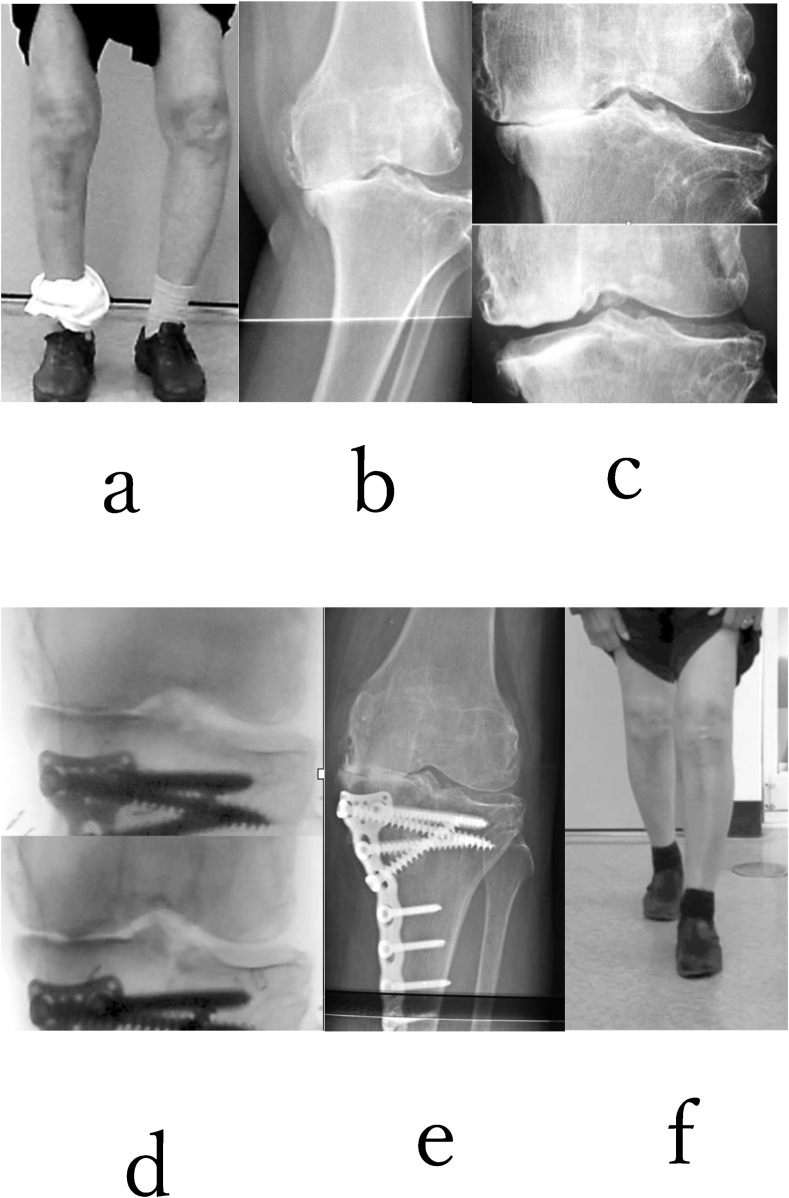
A 72-year-old woman with grade 4 (K–L) varus-type knee osteoarthritis in her both knees was treated with TCVO. Before TCVO, she could not transport without two crunches. At first, right TCVO was devised, and she could somehow walk without crutches. (a,b) Next, left TCVO was devised. She could return to work as a farmer. (e,f) The JOA score was from 30 points to 95 points after both TCVO. %MAD (left knee) was from −30% to 50% after TCVO. The range of motion of both knee joints was maintained (0° extension, 120° flexion) after TCVO. KJIA (knee flexed) was from 11.9° (c) to 1°(d) (left knee).

(Fig. 4),(Fig. 5) (Fig. 6).

## 4 Discussion

HTO is believed to treat varus alignment of the knee associated with medial mechanical axis deviation, leading to medial compartment overload and subsequent OA. HTO is contraindicated in patients with severe OA of the medial compartment.^2^, ^3^, ^4^, ^10^ Kettelkamp et al. reported that excessive bone loss from one plateau prohibits weight-bearing on both plateaus after osteotomy and results in an unstable knee. They called this situation the teeter effect because tibiofemoral contact shifts or teeters from one side to the other depending on the relationship between the center of gravity and the center of the knee. They concluded that excessive bone loss prevents two plateau weight-bearing after osteotomy introduces a teeter effect and is a contraindication. They pointed out the importance of simultaneous two-plateau weight bearing after the osteotomy for good clinical results. Several authors have reported poor results following HTO in patients with knee joint instability.^15^, ^17^ Recent studies have described HTO in cases with combined instability and varus malalignment using simultaneous or staged ligament reconstruction procedures alongside HTO.^1^, ^11^, ^13^ Combined ligamentous instability and malalignment remains a challenging problem for knee surgeons; however, ligament reconstruction creates donor site morbidity and has its own set of risks and complications.

Using a TCVO, adequate correction is indicated by parallelism of the lateral tibial plateau and a line tangential to the most distal point of the convexity of the lateral femoral condyle on an AP view using an image intensifier. The critical factor in assessing the adequacy of correction is the stability of the knee joint. The laminar spreader was used to gradually open the wedge until both tibial plateaus come in contact with the femoral condyles, and the knee becomes stable to varus-valgus stress (Fig. 2) The TCVO produces an increase in the contact area between the proximal tibial articular surface and the distal femoral articular surface by restoring congruency to the joint, thereby improving bony stability. This particularly prevents varus/valgus instability during gait and therefore eliminates varus thrust. By restoring congruency to the bony articulation, force is distributed across a wider area within the knee, leading to a decrease in the pressure per unit area applied to the knee joint. We hypothesized that an increase in bony stability contributes to a reduction in pain through these mechanisms.

In addition, adequate correction is indicated by parallelism of the lateral tibial plateau and a line tangential to the most distal point of the convexity of the lateral femoral condyle on an AP view using the image intensifier. A critical factor in assessing the adequacy of correction is the stability of the knee joint. A laminar spreader was used to gradually open the wedge until both tibial plateaus come in contact with the femoral condyles, and the knee becomes stable to varus-valgus stress. We concluded that the most important concepts of TCVO are the secure contact of both tibial plateaus and the femoral condyles and the stable knee after correction. Therefore, we never used the %MA, MPOA, MPA, and LPOA for the correction of TCVO, as well as MPTA. These radiologically measured values are the results of TCVO correction, and %MA was uncontrollable in TCVO because the correction of the intra-articular deformity in varus knee OA was not used in TCVO.

In contrast, an extra-articular HTO transfers the mechanical axis of the limb laterally and reduces the load on the medial compartment. HTO, therefore, only corrects alignment and has no effect on joint congruity or ligament stability. The relationship between the medial and lateral tibial plateaus did not change relative to each other and only changed relative to the distal femur in the coronal plane. HTO is an alignment correction surgery, and TCVO is a joint stabilization surgery.

Recently, some authors have reported on TCVO. However, they corrected varus knee OA using %MA.^5^, ^6^ The TCVO in these papers is an alignment correction surgery and completely different from the original TCVO because the original TCVO was never corrected using %MAD and Paley's indices.^12^

## 5 Conclusion

TCVO is indicated for all grades of varus knee OA in patients of all ages. The fundamental principle underlying TCVO is stabilization of the knee joint through the intra-articular correction of an intra-articular deformity. This intra-articular osteotomy improves both bony and soft tissue instability and creates a congruent joint with a load distributed over a larger surface area. TCVO stabilizes varus knee OA without the need for separate ligament reconstruction. Clinical results confirm that following TCVO, patients’ pain is improved, and they are able to return to heavy manual labor and sporting activity.

## Declaration of competing interest

The authors report no conflict of interest with the content of this manuscript.
